# Propofol administration to the maternal-fetal unit improved fetal EEG and influenced cerebral apoptotic pathway in preterm lambs suffering from severe asphyxia

**DOI:** 10.1186/s40348-015-0016-4

**Published:** 2015-03-10

**Authors:** Matthias Seehase, Ward Jennekens, Alex Zwanenburg, Peter Andriessen, Jennifer JP Collins, Elke Kuypers, Luc J Zimmermann, Johan SH Vles, Antonio WD Gavilanes, Boris W Kramer

**Affiliations:** Department of Paediatrics, Maastricht University Medical Center, P. Debyelaan 25, 6202 AZ Maastricht, The Netherlands; School of Mental Health and Neuroscience; School of Oncology and Developmental Biology, Maastricht University, and European Graduate School of Neuroscience (EURON), P. Debyelaan 25, 6202 AZ Maastricht, The Netherlands; Department of Clinical Physics, Maasstad Hospital, Postbus 9100, 3007 AC Rotterdam, The Netherlands; Department of Biomedical Technology, Maastricht University, Faculty of Health, Medicine and Life Sciences, School of Cardiovascular Diseases, P. Debyelaan 25, 6202 AZ Maastricht, The Netherlands; Department of Paediatrics, Máxima Medical Center, De Run 4600, 5504 DB Veldhoven, The Netherlands; Regenerative Medicine Program, Sprott Centre for Stem Cell Research at the Ottawa Hospital Research Institute, 725 Parkdale Ave, Ottawa ON K1Y 4E9, Ontario Canada; Department of Neurology, Maastricht University Medical Center, Faculty of Health, Medicine and Life Sciences, School of Mental Health and Neuroscience, Maastricht University, and European Graduate School of Neuroscience (EURON), P. Debyelaan 25, 6202 AZ Maastricht, The Netherlands; Department of Pediatric Cardiology and Intensive Care Medicine with Neonatology, University Hospital, Georg-August-University, Robert-Koch-Str. 40, D 37099 Göttingen, Germany

**Keywords:** Asphyxia, Propofol, Preterm, Neuroprotection, Lamb, Neonatal resuscitation

## Abstract

**Background:**

Term and near-term infants are at high risk of developing brain injury and life-long disability if they have suffered from severe perinatal asphyxia. We hypothesized that propofol administration to the maternal-fetal unit can diminish cerebral injury in term and near-term infant fetuses in states of progressive severe asphyxia.

**Methods:**

Forty-four late preterm lambs underwent total umbilical cord occlusion (UCO) or sham treatment *in utero*. UCO resulted in global asphyxia and cardiac arrest. After emergency cesarean section under either maternal propofol or isoflurane anesthesia, the fetuses were resuscitated and subsequently anesthetized the same way as their mothers.

**Results:**

Asphyctic lambs receiving isoflurane showed a significant increase of total and low-frequency spectral power in bursts indicating seizure activity and more burst-suppression with a marked increase of interburst interval length during UCO. Asphyctic lambs receiving propofol showed less EEG changes. Propofol increased levels of anti-apoptotic B-cell lymphoma-extra large (Bcl-xL) and phosphorylated STAT-3 and reduced the release of cytochrome c from the mitochondria and the protein levels of activated cysteinyl aspartate-specific protease (caspase)-3, -9, and N-methyl-d-aspartate (NMDA) receptor.

**Conclusions:**

Improvement of fetal EEG during and after severe asphyxia could be achieved by propofol treatment of the ovine maternal-fetal unit. The underlying mechanism is probably the reduction of glutamate-induced cytotoxicity by down-regulation of NMDA receptors and an inhibition of the mitochondrial apoptotic pathway.

## Background

Perinatal asphyxia has been associated with severe neurological and psychiatric sequelae resulting from injury in the basal ganglia/thalamus and cerebral cortex [1]. Despite the fact that therapeutic hypothermia is now standard care and improved the outcome in term-born infants after mild and moderate perinatal asphyxia [2], the incidence of neurological disabilities related to perinatal brain damage due to severe asphyxia has not decreased significantly in Western countries over the last decades [3]. In the clinical setting, after resuscitation of an infant with severe birth asphyxia, the emphasis is on supportive therapy [4]. Brain injury after perinatal asphyxia often develops with delayed clinical onset, opening a therapeutic window. It has been shown previously that apoptosis is the leading feature of cerebral cell loss in human fetuses suffering from asphyxia-related neonatal brain injury [5]. Many environmental and therapeutic agents initiate apoptotic cell death by inducing the release of cytochrome c from the mitochondria [6], which activates cysteinyl aspartate-specific protease (caspase)-9, an initiator caspase, and finally recruits the effector caspase-3. This initiates the caspase cascade that is responsible for the execution phase of apoptosis [6]. This pathway is controlled by B-cell lymphoma-extra large (Bcl-xL) and phosphorylated signal transducer and activator of transcription-3 (pSTAT-3) [7].

Currently, the only clinically established approach to reduce cell loss in asphyxia is moderate hypothermia [2]. In adult patients undergoing surgical procedures and clinical situations where protecting the CNS is a priority (cardiopulmonary bypass, subarachnoid hemorrhage, stroke, and postcardiac arrest resuscitation), the choice of anesthetic drug is essential [8]. Except for xenon [9], anesthetic agents have not been tested for their potential to protect the developing brain. One of the most promising anesthetic agents in mediating neuroprotection in hypoxic insults in adults is propofol [10]. Apart from its multiple anesthetic advantages, the short-acting intravenous anesthetic agent exerts a number of non-anesthetic effects. Propofol has proved to be able to reduce glutamate and N-methyl-d-aspartate (NMDA) receptors responses related to cytotoxicity in the brain [10]. Further on, it decreased the secretion of proinflammatory cytokines, impaired neutrophil chemotaxis, phagocytosis, and production of reactive oxygen species (ROS) [11,12]. This is of importance as it may be a way to interrupt the neuroinflammatory cascade characterized by astrocyte and microglial activation, cytokine release, and ROS formation following hypoxia.

Based on these considerations, we hypothesized that propofol may reduce neonatal brain injury resulting from severe asphyxia and fetal cardiac arrest *in utero* if propofol is administered to the maternal-fetal unit already before birth and continued postnatally.

To this purpose, we established a perinatal global asphyxia and resuscitation model in near-term sheep in which we evaluated brain function by EEG and the immunomodulatory and apoptotic changes by assessing several brain markers in the asphyxiated offspring of pregnant ewes who were sedated with either propofol or with isoflurane.

## Methods

### Animals

The study was approved by the Animal Ethics Research Committee, Maastricht University, The Netherlands. Texel ewes were date-mated, and the fetuses were randomized for umbilical cord occlusion (UCO) and sedation. Observations were made in 44 late preterm fetal sheep of both genders at mean (range) gestational age of 133 or 134 days (term 150 days). Eighteen sheep fetuses were subjected to total UCO *in utero*, and 18 fetal sheep served as sham controls (Table 1). In each group, about half of the pregnant ewes and their offspring were sedated with propofol and the other ones with isoflurane. Eight lambs were euthanized directly after preterm delivery and served as gestational age (GA) controls.

**Table 1:** **Numbers of lambs per group, gender, body weight, times of complete umbilical cord occlusion (UCO), and pH of cord blood**

	**GA control**	**Propofol control**	**Propofol asphyxia**	**Isoflurane control**	**Isoflurane asphyxia**
Included fetal lambs	8	11	10	7	8
Female/male	4/4	3 / 8	6 / 4	4 / 3	2 / 6
Body weight [kg]	3.59 ± 0.14	3.57 ± 0.15	3.86 ± 0.15	3.57 ± 0.15	3.51 ± 0.15
Exact time of MABP < 30 mmHg during UCO [min]	-	-	2.0 ± 0.0	-	2.0 ± 0.0
Mean time of UCO [min]	-	-	10.7 ± 0.3	-	11.3 ± 0.6
Chest compressions during CPR: yes/no	0/8	0/11	9/1	0/7	8/0
Mean duration of chest compression [min]	0	0	9.0 ± 0.3	0	9.0 ± 0.4
Adrenalin during CPR: yes/no	0/8	0/11	10/0	0/7	8/0
Total dose of adrenalin [μg/kgBW]	0	0	29 ± 10	0	32 ± 12
pH before birth (and UCO)	7.26 ± 0.01	7.26 ± 0.01	7.26 ± 0.01	7.26 ± 0.01	7.26 ± 0.01
pH after birth	-	7.10 ± 0.03	6.91 ± 0.03*	7.07 ± 0.02	6.84 ± 0.01*
pH after 8 h of intensive care	-	7.19 ± 0.05	7.21 ± 0.08	7.16 ± 0.11	7.06 ± 0.14

All data are given as absolute values or mean ± SEM. An asterisk (*) means significant difference with *p* < 0.01 versus corresponding control group. MABP, mean arterial blood pressure; UCO, umbilical cord occlusion; CPR, cardiopulmonary resuscitation.

### Experimental protocol

**Figure 1:**
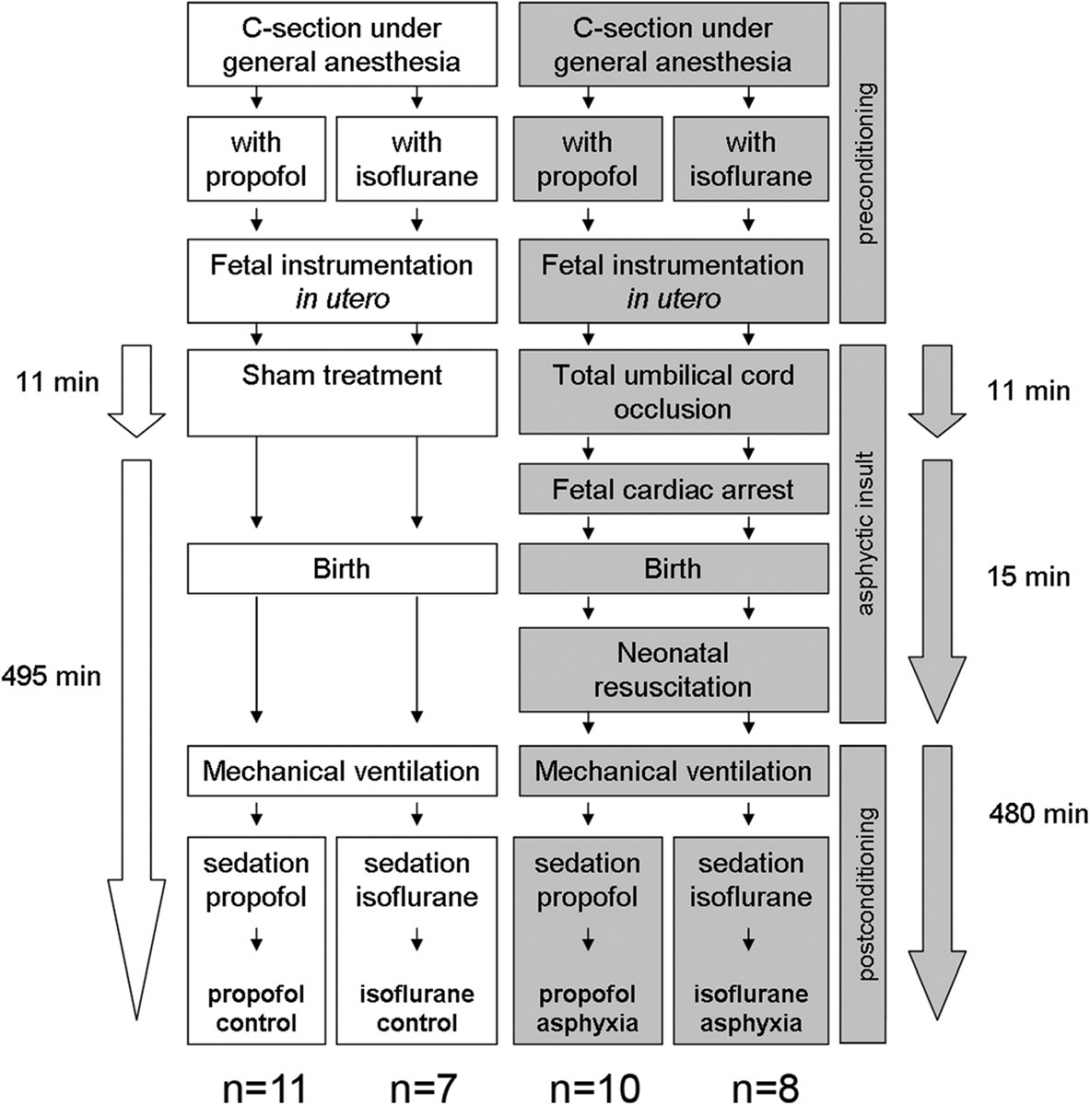
**Study design and time course of experiments.** Lambs were born at 89% of gestation. The ewes carrying the gestational age control group were euthanized and their offspring was delivered by cesarean section and subsequently euthanized. The ewes of the other groups underwent the cesarean section while being sedated with the same anesthetic as their offspring later on intensive care unit. Two groups were exposed to an average time of total umbilical cord occlusion (UCO) of 11 min. The cutoff for the occlusion was a MABP below 30 mmHg for exactly 2 min. This protocol always led to severe bradycardia (HR < 30/min) or even cardiac arrest.

We used the same experimental protocol as published previously [12]. In brief, the pregnant ewes were intubated and general anesthesia was maintained with isoflurane (1% to 2%) or propofol (25 mg/kg/h) during the cesarean section. Both anesthesia types were supplemented by continuous remifentanil infusion (3 μg/kg/min). After a lower midline incision, the fetus was carefully extracted through a small incision of the uterus. The fetus was instrumented with an endotracheal tube and an arterial catheter in the femoral and umbilical artery and a venous catheter in the external jugular vein. The umbilical cord was gently extracted through the incision of the uterus, and a vascular occluder (OC16HD, 16 mm, IN VIVO METRIC, Healdsburg, California, USA) was placed around the umbilical cord. The umbilical cord was occluded until the mean arterial blood pressure (MABP) dropped below 30 mmHg. From that time point onwards, the occlusion was continued for exactly 2 min (Figure 1). After these 2 min, occlusion was stopped regardless of whether the lambs suffered from bradycardia only or from cardiac arrest. After the end of the occlusion, the lambs stayed *in utero* for 20 s experiencing reperfusion. Then, they were delivered, the umbilical cord was cut, and resuscitation was started while the fetus was brought to an open, heated incubator (IW930 Series CosyCot™ Infant Warmer, Fisher & Paykel Healthcare). The incubator maintained the lamb’s body temperature of 38.5°C. When the lambs were delivered and when their umbilical cord was cut, they were initially ventilated with a resuscitator bag via the endotracheal tube with room air (60/min). Heart massage was started with around 120 compressions per minute. The fetus was brought to the incubator with one medical operator doing the heart massage while the other one was ventilating the lamb. After arrival at the incubator, the lamb was connected to a ventilator Servo 900C (Siemens-Elema, Solna, Sweden), put into plastic foil to prevent cooling effects, and adrenaline was administered via the venous catheter in augmented doses from 30 μg, to 60 μg, to 0.1 mg. In addition, a volume bolus of ringer lactate of 10 ml/kg BW was administered after the first shot of adrenaline. The lambs were administered with pressure-regulated ventilation using a ventilator with initial settings as follows: FiO_2_ = 1, positive end-expiratory pressure, 5 cmH_2_0, peak inspiratory pressure 30 cmH_2_0, frequency 60/min, and I:E 1:2. Thereafter, inspiratory pressure was adjusted to achieve a targeted volume of 3.0 L/min and a P_a_CO_2_ of 35 to 45 mm Hg. The ventilation and sedation was continued for 8 h after delivery. The sedation was maintained either with isoflurane (0.5% to 1.0%) or propofol (1 to 3 mg/kg/h) and supplemented with remifentanil (3 μg/kg/min) in both groups. All cord-occluded lambs developed a spontaneous HR (>150/min) and a sufficient MABP (>50 mmHg) within 10 min after starting resuscitation with the exception of two lambs, which were excluded from the study.

The 18 sham-treated fetuses were prepared *in utero* with the same catheters and endotracheal intubation as the study groups. Eleven minutes (mean time of occlusion in study group as determined in advance in pilot experiments) after the end of the instrumentation, they were delivered, weighed, sedated, and connected to pressure-controlled ventilation (see above).

At 8 h after delivery, the lambs were euthanized by an i.v. injection of T61® (Veterinaria AG, Zürich, Switzerland).

The pregnant ewes carrying the GA control lambs were euthanized using an injection of T61®. Their offspring was delivered surgically immediately thereafter and were also directly euthanized using an injection of T61® without any previous catheterization or ventilation before.

After euthanasia, the brains of all lambs were immediately removed from the skull and separated at the midline. From one hemisphere, tissue samples were taken and snap-frozen in liquid nitrogen. Tissue was subsequently stored at −80°C.

### Electroencephalogram

Two bipolar pairs of electroencephalogram (EEG) silver ball electrodes (AS633-5SSF, Cooner wire Co., Chatsworth, CA, USA) were placed subcutaneously over the left and right parasagittal parietal cortex (5 and 15 mm anterior to bregma and 10 mm lateral), with a reference electrode placed over the occiput.

The EEG signal was sampled at 250 Hz and stored on hard-disk for off-line analysis. Data were filtered using a 0.5-Hz high-pass and 30-Hz low-pass fourth-order Butterworth filter. EEG signal with an amplitude >1,000 μV was considered an artifact and removed from analysis (1% of data). After filtering, background analysis of the EEG was performed using an amplitude- and time-threshold-based algorithm [13,14]. Burst activity was defined as an epoch with an amplitude >15 μV and a duration of >1 s in both channels. Interburst intervals were defined as epochs with an amplitude <15 μV and a duration >3 s in both channels [13]. Segments not meeting above criteria were classified as undefined [13]. Using this approach, the algorithm detected burst, five interburst interval, and undefined segments in 47%, 28%, and 25% of cases, respectively.

Subsequently, spectral analysis was performed using Matlab® (The MathWorks, Massachusetts, USA). Data was divided into half-overlapping 4-s segments around the center of each burst. Each segment was multiplied by a Hamming window before Fourier transform. The frequency spectrum was determined for each segment and was divided in δ1 (0.5 to 1 Hz), δ2 (1 to 4 Hz), θ (4 to 8 Hz), α (8 to 13 Hz), and β (13 to 30 Hz) band. Likewise, spectral analysis was performed for the interburst intervals.

Three aspects of the transformed signal were calculated: (a) absolute power (defined as the integral of all powers within the frequency band 0.5 to 30 Hz, expressed in μV^2^), (b) relative power (defined as the ratio of absolute band power to total power of all bands, expressed in percentage), (c) spectral edge frequency (SEF defined as the frequency that delimits 90% of the power between 0.5 and 30 Hz). Finally, the obtained parameters were averaged over the left and right channel to obtain median burst frequency parameters per measurement.

In addition, the raw EEG signal was converted in an amplitude-integrated EEG (aEEG) using the software of the NicoletOne™ device (Viasys Healthcare, Conshohocken, PA, USA). The aEEG is a technique in which the EEG signal is expressed in a simplified and time-compressed manner [15]. In the neonatal intensive care unit (NICU), the aEEG has shown to be a very useful tool for long-term bed-side monitoring of neonatal seizures and background patterns [16].

### Western blotting

Western blots were performed as described previously [12,17]. Briefly, the whole frozen frontal cortex was homogenized in ice-cold RIPA buffer (R0278-50ML, Sigma-Aldrich Corp., St. Louis, MO, USA) containing Halt Protease and Phosphatase Inhibitor Cocktail, EDTA-free (100×) (Thermo Fisher Scientific Inc., Rockford, IL 61105 USA). The samples were then centrifuged at 500×*g* for 20 min at 4°C to remove cellular debris. Protein content in the supernatant was determined using the Micro BCA Protein Assay Kit (Thermo Fisher Scientific) with BSA as the standard.

The following dilutions for antibodies were used: 1:1,000 for monoclonal anti-β-actin clone AC-1 (Sigma-Aldrich Catalog Number A5441), anti-cleaved-caspase-3 (Asp175) (Cell Signaling Technology, Inc., Danvers, MA, USA), anti-cleaved-caspase-9 (Novus Biologicals, Littleton CO, USA), anti-Bcl-xL (Cell Signaling Technology, Inc., Danvers, MA, USA), anti-phospho-Akt (Cell Signaling Technology, Inc., Danvers, MA, USA), anti-pSTAT-3 (Cell Signaling Technology, Inc., Danvers, MA, USA), and anti-NMDA receptor (Cell Signaling Technology, Inc., Danvers, MA, USA). As secondary antibody IRDye® 680 conjugated goat (polyclonal) anti-rabbit IgG, IRDye® 800 conjugated goat (polyclonal) anti-rabbit IgG, IRDye® 680 conjugated goat (polyclonal) anti-mouse IgG, and IRDye® 800 conjugated goat (polyclonal) anti-mouse IgG (LI-COR, Lincoln, Nebraska, USA) were used in a dilution of 1:6,000. The blots were analyzed using the LICOR Odyssey Infrared Imaging System, and the signals of the target proteins were normalized to the signal of β-actin which acts as a house-keeping protein. Images were acquired using Adobe Photoshop CS6 software.

### Cytochrome-c-releasing apoptosis assay kit

Cytochrome c release was determined using the cytochrome-c-releasing apoptosis assay kit (BioVision, Mountain View, CA, USA) according to the manufacturer’s protocol.

### Lipid peroxidation assay

For brain tissue analysis, we used the ALdetect (MDA-Specific) Lipid Peroxidation Assay Kit (ALdetect (MDA-Specific) Lipid Peroxidation Assay Kit BML-AK-171, Enzo Life Sciences International, Inc., Plymouth Meeting, PA, USA) according to the manufacturer’s manual.

### Data analysis

Data with a normal distribution are expressed as a mean ± SEM; otherwise, data are expressed as a median and interquartile range (IQR). Physiological variables were analyzed using two-way ANOVA. All other data were analyzed by the Mann-Whitney-Wilcoxon test. A *p* value <0.05 was considered significant. All statistical analyses were performed using the statistical software GraphPad Prism 5.0.

## Results

The lambs had similar gestational ages of 133.7 ± 0.16 days and similar birth weights of 3.6 ± 0.1 kg. All animals developed a severe bradycardia with a frequency of <30/min or a cardiac arrest upon reperfusion. Gender distribution, body weights, and pH and resuscitation details are given in Table 1. The temperature of all lambs was kept during the whole experiment between 38.0°C and 38.5°C. Heart rate and blood pressure did not differ between groups as published previously [12].

### EEG

#### Baseline before UCO

**Figure 2:**
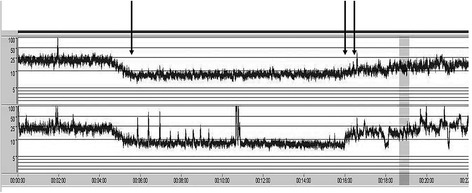
**Bilateral amplitude-integrated EEG before, during, and after occlusion of the umbilical cord.** The *x*-axis shows time in minutes. The *y*-axis shows the amplitude of the signal and is displayed semi-logarithmic: linear 0 to 10 μV and logarithmic 10 to 100 μV. The vertical arrow (left) represents the onset of occlusion. Before occlusion, the lower margin amplitude is 20 to 25 μV. Within 1 min, the lower margin amplitude decreased to values <10 μV. After occlusion (double arrow), the lower margin amplitude increased to pre-occlusion values.

**Figure 3:**
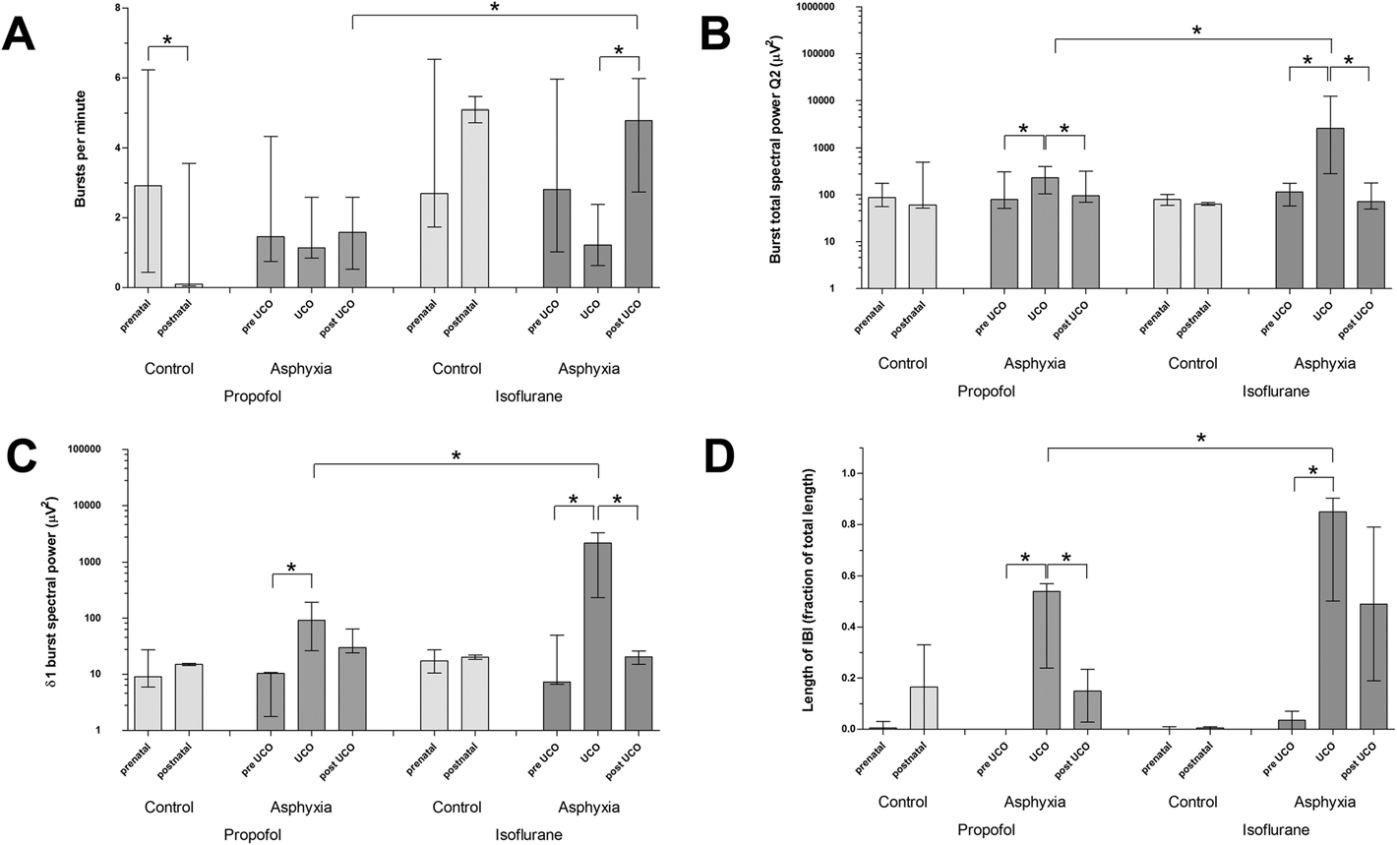
**EEG analysis.** Pre- and postnatal EEG of control groups and EEG before, during, and after UCO in asphyxia groups. Depicted are **(A)** the median burst per minute, **(B)** the median total spectral power, **(C)** the relative spectral power for δ1, and **(D)** the interburst interval length as median and interquartile range. Significant differences (*p* < 0.05) are marked by (*). UCO, umbilical cord occlusion; IBI, interburst interval.

Figure 2 illustrates the aEEG trace before, during, and after the UCO. During baseline condition, the lower margin amplitude is >10 μV and the upper margin amplitude is <25 μV, consistent with a normal background pattern of the brain. During baseline conditions, the median bursts per minute, the median total spectral power, the relative spectral power estimates, and the median SEF values were not different between the four experimental groups (Figure 3).

#### Changes of burst activity

**Figure 4:**
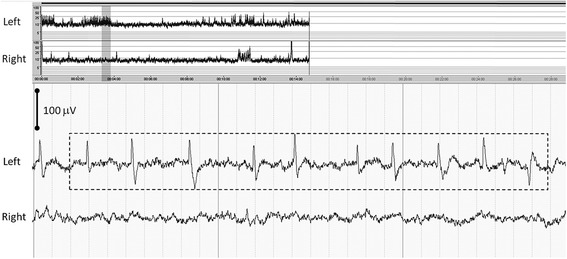
**EEG during the postocclusion phase.** A 15-min epoch of amplitude-integrated EEG during the postocclusion phase (*top*, *x*-axis shows time in minutes, *y*-axis shows the amplitude of the signal and is displayed semi-logarithmic: linear 0 to 10 μV and logarithmic 10 to 100 μV) shows a slight increase of the lower margin amplitude and increase of bandwidth, indicated by the shaded dark gray vertical band. Illustration of the accessory bilateral EEG during the shaded vertical band of the aEEG (*bottom*, time resolution 1 s/dotted vertical line, sensitivity indicated by vertical stick) showing repetitive sharp waves every 2 to 3 s, suggestive of seizure activity in the left hemisphere. The rectangular box indicates the period over which spectral analysis is performed and shows a relative delta power of 84%.

During UCO, the aEEG pattern rapidly changed with upper margin amplitude values <10 μV, consistent with a low-voltage pattern (Figure 2). In isoflurane-treated lambs, we observed a 60% decrease in burst per minute during UCO, whereas in propofol-treated lambs a much smaller change of 20% was observed. In the post-UCO phase, we observed significantly more burst activity in the isoflurane lambs than in the propofol-treated lambs (median burst/minute 4.8 vs. 1.6, *p* < 0.05) (Figure 3A). Burst total spectral power during UCO increased more in isoflurane-treated lambs than in propofol-treated lambs (Figure 3B). Of the relative spectral power measures, δ1 and δ2 were particularly responsible for the increase of burst spectral power in isoflurane-treated lambs (Figure 3C). The increase of burst spectral power and relative delta activity was associated with characteristic repetitive patterns of sharp waves of the raw EEG, indicating seizure activity (Figure 4).

We observed no significant change in SEF during UCO or postnatally between isoflurane- and propofol-treated lambs.

#### Changes of interburst interval

We found a significant increase in interburst interval length during UCO, which was significantly greater in isoflurane- than propofol-treated lambs (Figure 3D). In the post-UCO phase, this difference became smaller but was still significant.

### Western blot analysis

#### N-methyl-d-aspartate-receptor (NMDA-R)

**Figure 5:**
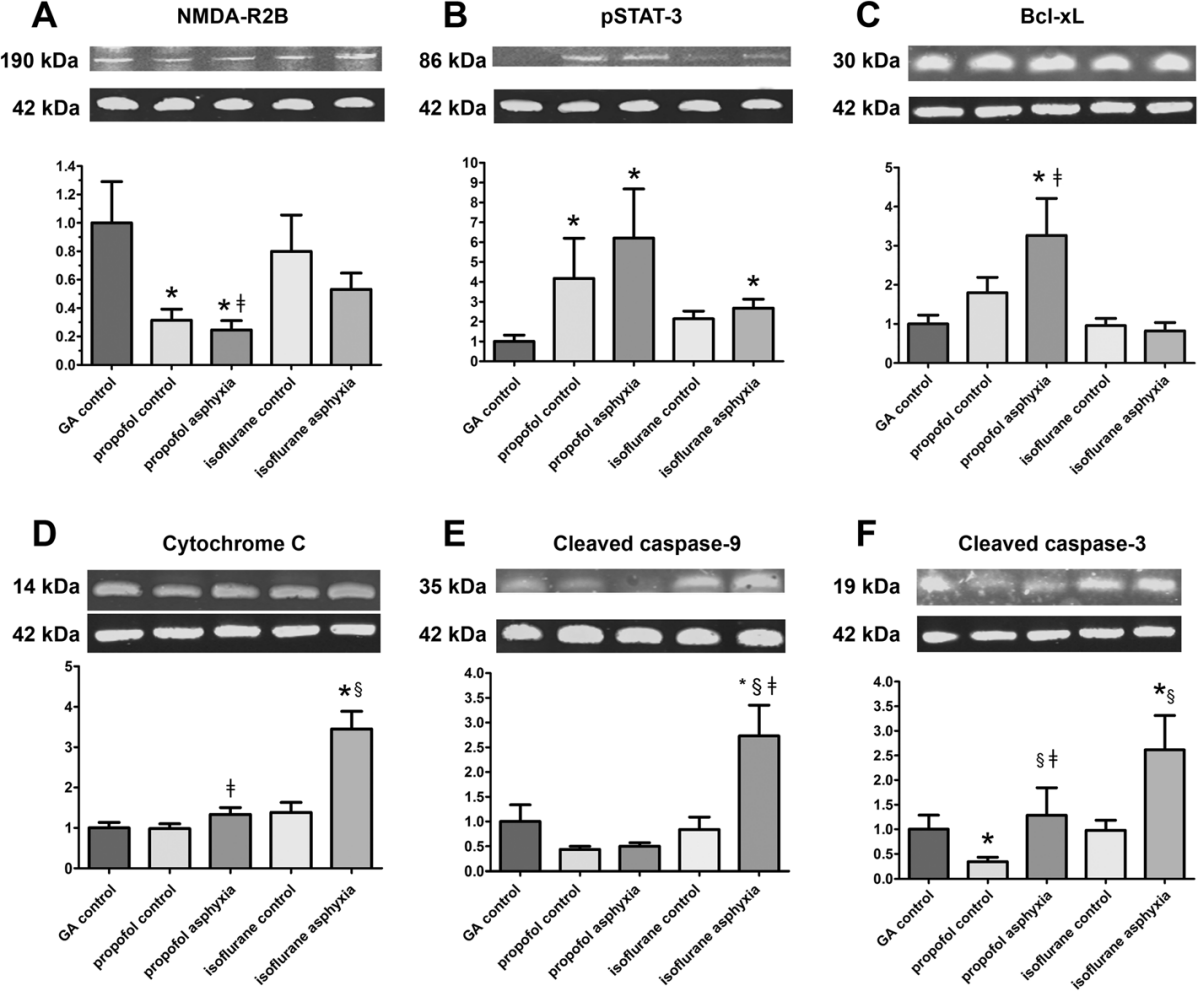
**Western blot in frontal cortex of fetal brain.** Western blot for **(A)** NMDA receptors, **(B)** pSTAT-3, **(C)** Bcl-xL, **(D)** cytochrome c, **(E)** cleaved caspase-9, and **(F)** cleaved caspase-3 in frontal cortex of fetal brain normalized to β-actin. Levels of the gestational age control group were set to 1 in all panels. Depicted are mean and SEM. Significant differences (*p* < 0.05) compared to gestational age control groups are marked by (*). Significant differences (*p* < 0.05) between asphyxiated and corresponding non-asphyxiated drug control group are marked by § and significant differences between asphyxiated propofol and asphyxiated isoflurane treated lambs are marked by ‡. NMDA-R, N-methyl-d-aspartate-receptor; GA, gestational age; pSTAT-3, phosphorylated signal transducer and activator of transcription-3; Bcl-xL, B-cell lymphoma-extra large; caspase, cysteinyl aspartate-specific protease.

In the propofol control group, the level of N-methyl-d-aspartate-receptor (NMDA-R) decreased to 31.5% ± 7.8% (*p* < 0.02) compared to the GA control group (Figure 5A). After prenatal asphyxia, NMDA-R levels decreased in propofol-treated lambs to 24.6% ± 6.6% (*p* = 0.04) compared to the GA controls and to 46.2% ± 21.7% (*p* < 0.05) when compared to the isoflurane asphyxia group.

#### Phosphorylated signal transducer and activator of transcription-3 (pSTAT-3, Tyr705)

Protein levels of pSTAT-3 increased in the propofol control group compared to the GA control group up to 417.8% ± 202.5% (*p* < 0.05; Figure 5B). In the propofol asphyxia group, pSTAT-3 increased to 620.8% ± 247.2% (*p* < 0.01) compared to the GA control group. After prenatal asphyxia, pSTAT-3 increased in isoflurane-treated lambs to 267.7% ± 45.5% compared to the GA controls (*p* < 0.02).

#### B-cell lymphoma-extra large (Bcl-xL)

In the propofol asphyxia group, Bcl-xL increased to 326.2% ± 94.8% (*p* = 0.03; Figure 5C) compared to the GA control group and to 397.0% (*p* < 0.02) compared to the isoflurane asphyxia group.

#### Cytochrome-c-releasing apoptosis assay

In the isoflurane asphyxia group, cytochrome c increased to 344.7% ± 43.9% compared to the GA control group (*p* < 0.01), to 249.4% (*p* < 0.01) compared to the isoflurane control group, and to 258.4% (*p* < 0.01) compared to the propofol asphyxia group (Figure 5D).

#### Cleaved caspase-9

In the isoflurane asphyxia group, cleaved caspase-9 increased to 273.1% ± 62.1% (*p* = 0.04) compared to GA, to 325.4% (*p* < 0.02) compared to the isoflurane controls, and to 545.7% (*p* < 0.01) in comparison to the propofol asphyxia group (Figure 5E).

#### Cleaved caspase-3

In the propofol control group, the protein level of cleaved caspase-3 decreased to 34.2% ± 9.0% (*p* < 0.04) compared to the GA control group (Figure 5F). Cleaved caspase-3 increased by 375.3% in the propofol asphyxia group compared to the propofol controls (*p* < 0.04). After prenatal asphyxia, cleaved caspase-3 increased in isoflurane-treated lambs by 266.7% compared to the isoflurane controls (*p* = 0.01), by 261.2% ± 69.9% (*p* < 0.05) compared to the GA control group, and up to 203.2% ±54.44% (*p* < 0.03) compared to the propofol asphyxia group.

### Lipid peroxidation assay

#### Malondialdehyde (MDA)

**Figure 6:**
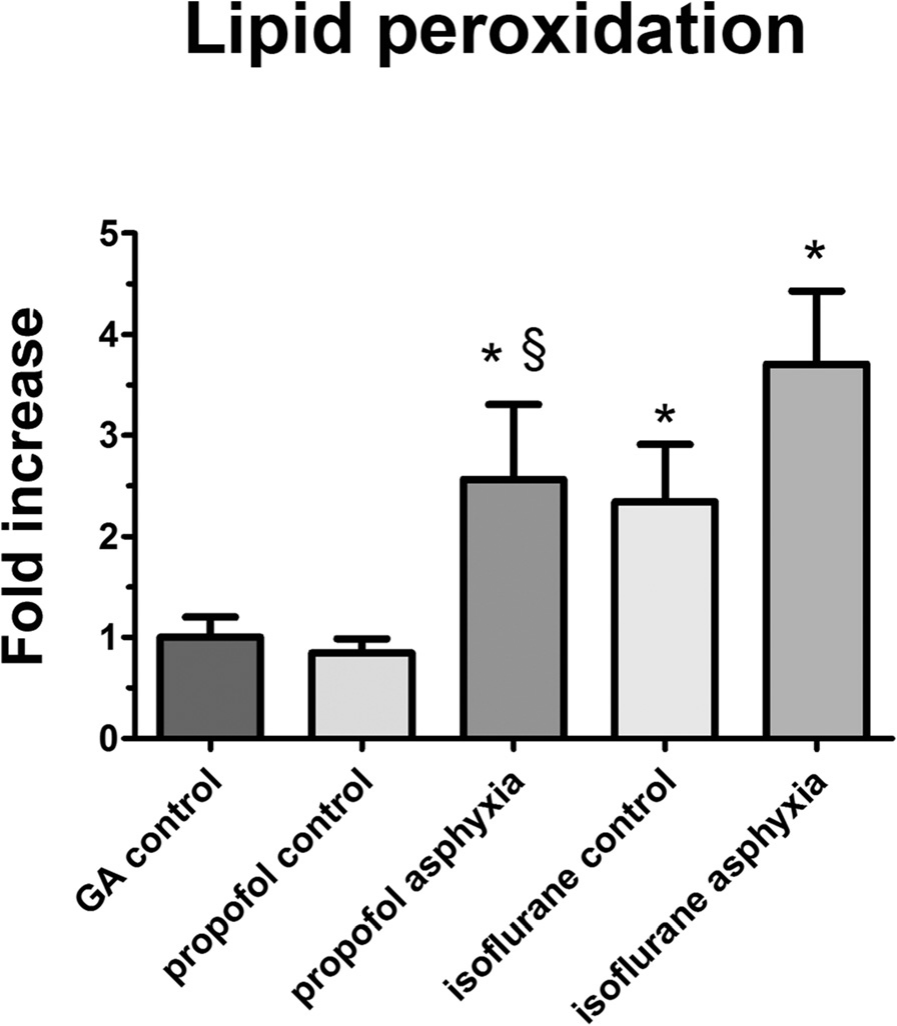
**Reactive oxygen species in cerebral fetal cortex.** Malondialdehyde (MDA) levels in cerebral fetal cortex as indication of lipid peroxidation due to reactive oxygen species (ROS) formation. Levels of the gestational age control group were set to 1. Depicted are mean and SEM. Significant differences (*p* < 0.05) compared to gestational age control groups are marked by (*). Significant differences (*p* < 0.05) between asphyxiated and corresponding not-asphyxiated drug control group are marked by §. GA, gestational age.

Malondialdehyde (MDA) increased by 302.9% in the propofol asphyxia group compared to the propofol controls (*p* < 0.01) and by 256.1% ± 74.4% (*p* = 0.04) compared to the GA control group (Figure 6). In the isoflurane control group, MDA increased by 234.0% ± 57.0% (*p* = 0.02) compared to the GA control group. After prenatal asphyxia, MDA increased by 370.4% ± 71.9% (*p* < 0.01) compared to the GA control group.

## Discussion

**Figure 7:**
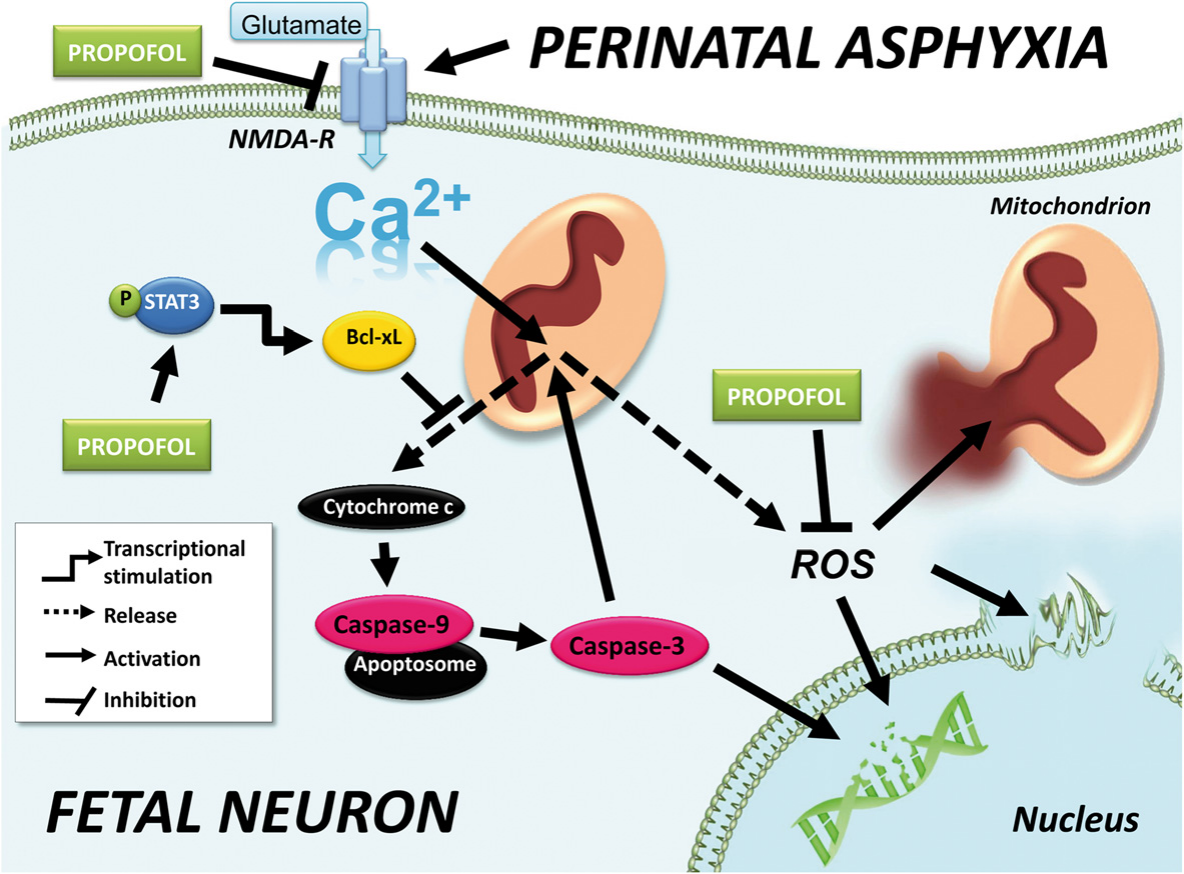
**Activation and inhibition of apoptotic pathways in the fetal ovine brain.** Propofol reduced expression of NMDA receptors and occurrence of reactive oxygen species (ROS). In addition, Propofol treatment resulted in activation of pSTAT-3 which inhibited release of cytochrome c from the mitochondria. Consequently, activation of caspases-9 and -3 was reduced. NMDA-R, N-methyl-d-aspartate-receptor; pSTAT-3, phosphorylated signal transducer and activator of transcription-3; Bcl-xL, B-cell lymphoma-extra large; caspase, cysteinyl aspartate-specific protease.

In this paper, we demonstrate molecular (Figure 7) and functional changes of the cerebrum of late preterm lambs in a unique experimental standardized sequence of severe asphyxia due to complete umbilical cord occlusion *in utero*, fetal cardiac arrest, emergency cesarean section, postnatal resuscitation of the fetus, and intensive care for the first 8 h after birth. The molecular and functional changes indicate that medication has the potential to improve fetal cortical function as assessed by amplitude-integrated EEG when the medication is administered already before birth to the maternal-fetal unit during cesarean section and postnatally to the baby on the NICU.

We observed that isoflurane administration to the maternal-fetal unit before and during the umbilical cord occlusion resulted in EEG changes with increased burst spectral power when compared to the propofol-treated and occluded group. The increased δ1 and δ2 spectral power burst activity may indicate seizure activity, as this is characteristically manifested by low-frequency high-voltage EEG waves [18,19]. This suggests that isoflurane is associated with more epileptogenic activity than propofol. Despite sedation, activity of clinical convulsions was observed in all animals that underwent UCO. In order to quantify this potentially biased clinical judgment, we used this standardized analysis. Quantification of the effects is extremely important in this new approach since seizures in the neonatal period can be extremely refractory to therapy [20]. The immature brain may be more susceptible to the epileptogenic effects of provoking stimuli, such as hypoxia [20]. Seizures may alter brain development resulting in epilepsy and neurologic impairment in later life, even in the absence of obvious infarction or other structural injury [20]. In this context, the smaller increase of (low-frequency) spectral power during bursts during UCO in our propofol-treated lambs may be considered beneficial. Moreover, the length of interburst interval, which is considered to be a predictive EEG marker of neonatal brain injury [21], was less increased during UCO in propofol-treated lambs compared to isoflurane controls. Hence, less burst-suppression of the EEG was recorded during propofol sedation. These changes can partly be explained by propofol, which has the potential to suppress seizure activity via inhibition of the NMDA receptor and modulation of the slow calcium ion channels [22]. A wide range of doses of propofol can suppress the interictal and ictal activity and abolish the epileptiform discharges, electrographic seizures, clinical seizures, and the status epilepticus inducing the burst-suppression pattern [22]. However, data on propofol-induced EEG changes in epilepsy patients are limited to a few small-size studies in adults [22], and it is not conclusive as to whether propofol activates or depresses EEG seizure activity in this patient population [23]. Meyer and colleagues studied the effects of propofol and its continuous infusion on the EEG in 25 pediatric epilepsy patients which were sedated to carry out MRI scans. In 16 out of 18 patients with epilepsy and documented spike-wave patterns prior to propofol sedation, a suppression of spike-wave patterns (>90% of baseline seizure activity) was demonstrated. In addition, no depression or augmentation of EEG amplitudes, a primary occurrence, or an increase in spike-wave patterns was seen [22,24]. Aside from EEG as a functional readout, we found that propofol treatment protected the auditory brainstem response (ABR) threshold as compared to isoflurane anesthesia in this model as published previously [25].

Not only does NMDA-receptor-mediated signaling influence seizure activity and auditory function, but the NMDA receptor is also involved in hypoxia-reperfusion injury. An overstimulation of NMDA receptors leads to an excessive Ca^2+^-influx and plays a major role in the series of potentially neurotoxic events [26]. One of these events is the Ca^2+^ overload of mitochondria, leading to the generation of ROS. Therefore, inhibition of the NMDA receptor, or preventing accumulation of ROS, would attenuate brain damage. A recent study by Sandoval et al. [27] provided data that even a down-regulation of NMDA receptors mediates neuroprotection in cortical cultures. Synaptic NMDA receptors have anti-apoptotic activity, whereas stimulation of extrasynaptic NMDA receptors results in a loss of mitochondrial membrane potential which is an early marker for glutamate-induced neuronal damage and cell death [28]. Blocking specifically extrasynaptic NMDA receptors may effectively prevent neuronal cell death in conditions associated with ischemia and glutamate toxicity [28]. The same group showed that the NMDA receptor NR2A subunits are localized primarily at synapses and promote cell survival whereas NR2B subunits are contained predominantly in extra-synaptic NMDA-Rs and promote cell death. Consistent with the concept that NR2B is involved in cell death, Sandoval et al. found that the selective pharmacological blockade of NR2B prevented cell death in cortical cultures [27]. In line with this, all propofol-sedated lambs in our study showed a down-regulation of the NMDA receptor subunit NR2B and better EEG features and less cerebral apoptosis compared with isoflurane-treated animals.

A potential concern in using anesthetic drugs in preterm and term newborn infants has come from animal studies. Anesthetics caused massive induction of apoptosis in the developing brain of newborn rodents [29]. However, considering the short gestation in rats (22.5 days) and mice (19.5 days), and their consequently faster brain development, compared to the relatively long human gestation (280 days), there may be significant differences in the effect of a pharmacological intervention aimed at crucial brain-development-mediating elements such as NMDA receptors. In addition, it has to be taken into account that in contrast to humans important steps such as white matter myelination occur in rodents after birth [30]. For these reasons, sheep appear to be an appropriate model since the ovine gestation period is 150 days with prenatal white matter myelination, which is much closer to the human development [31]. In addition, sheep have been already extensively used to study the effects of fetal and perinatal asphyxia [12,25]. Apoptosis is not only a possible consequence of exposure to anesthetic drugs but is the leading feature of cerebral cell loss in asphyxia-related neonatal brain injury [5]. During apoptosis, the permeabilization of the mitochondrial outer membrane allows the release of cytochrome c, which induces caspase activation to orchestrate the death of the cell. After loosing their transmembrane potential (ΔΨm), the mitochondria generate ROS which likely contributes to the dismantling of the cell. Cytochrome c release activates caspase-9 which in turn initiates the cleavage of caspase-3. Caspase-3 is the most effective apoptotic regulator because it plays a decisive role in the occurrence of apoptosis by acting as the effector caspase which determines the incidence of apoptosis [6]. In our study, cytochrome c release from the mitochondria and subsequent activation of caspase-9 and caspase-3 was unchanged in isoflurane-sedated control lambs compared to gestational age controls suggesting no neurotoxic effect due to isoflurane administration over 8 h. Surprisingly, caspase-3 activation even decreased in propofol-treated control lambs compared to gestational age controls and isoflurane-treated ones. Whereas a reduction in caspase-3 activation and thereby reduction of apoptosis may be beneficial in neurons under uncontrolled hypoxic conditions, an exogenously caused reduction in apoptosis in the healthy developing brain may be harmful because apoptosis is a vital mechanism in the formation process of neuroplasticity [32].

After UCO, cardiac arrest, successful resuscitation and 8 h of sedation with isoflurane, both caspase-3 and -9 activation and cytochrome c release increased around threefold compared to the normal situation at this time point in gestation. But after severe asphyxia, there was no increase in cytochrome c release and subsequently, caspase-3 and -9 showed no activation if lambs were treated with propofol, suggesting less to no induction of the mitochondrial apoptotic pathway.

A possible explanation for our findings was given by Li et al. [33]. His group provided evidence that propofol anesthesia during cerebral ischemia led to activation of Bcl-xL. Bcl-xL inhibits caspase-3 to influence mitochondrial function without access to the intermembrane space, since Bcl-xL blocks mitochondrial membrane permeabilization. Consistent with this, Bcl-xL levels were increased in fetal brains after propofol anesthesia in our experiment, suggesting that indeed propofol has an inhibitory effect on caspase-3 activation in the late preterm fetus, too. Previous studies have shown that activation of pSTAT-3 is required for expression of Bcl-xL [34]. In our experiments, propofol led to increased phosphorylation of STAT-3 whereas isoflurane treatment did not. These data support the concept that pSTAT-3 and Bcl-xL mediate the cytochrome c/caspase-3 apoptotic process not only in adult but also in cells of the fetal frontal cortex and that they can be activated by propofol.

Cytochrome c release and the activation of caspases feedback on the permeabilized mitochondria increasing mitochondrial damage and ROS generation [6]. ROS-mediated lipid peroxidation and DNA damage then appears to be the final common pathway for neuronal cell death [26]. The brain of term- and near-term-born infants seems particularly vulnerable to ROS formation and the resulting oxidative injury after asphyxia because of immature scavenging mechanisms and a relative abundance of iron that acts as a catalyst for the formation of free radicals [6]. One pathway by which propofol exerts its cell-protective properties in adults is the reduction of the occurrence of ROS after ischemia [10]. In experimental settings, it has been shown that activation of caspase-3 resulted in significant ROS production. The increase was inhibited by Bcl-xL, probably via inhibition of the caspase-activated permeabilization of the mitochondrial outer membrane as discussed above. Therefore, it is likely that the caspase-mediated disruption of respiratory chain complex I and II function contributes to high ROS production during apoptosis. This would account for the effect of caspase inhibition on apoptosis-associated ROS generation we observed in our experiment. The slightly reduced lipid peroxidation in our propofol-treated lambs suggest that the scavenging properties of propofol and its inhibitory effect on caspase-3 cleavage may play a minor role in mediating protection in the premature brain. Consistent with this notion, it has been demonstrated in human newborns that the degree of lipid peroxidation represents a valid predictive marker for birth-asphyxia-related morbidity and mortality [35].

One limitation of our study is that we only studied one time point in gestation, namely late preterm sheep with a gestational age of 133 days. The fetal brain matures particularly fast during this final third part of gestation, in both sheep and humans. Observations made at one time point cannot be easily extrapolated to earlier or later time points of gestation [36]. Despite the high similarities between ovine and human brain development, one has to be careful in extrapolating our data to the clinical situation in the hospital. An additional limitation is that we administered anesthesia to the lambs for 8 h whereas term and near-term infants with complications as birth asphyxia are only getting a full anesthesia if they undergo surgery, e.g., because of necrotizing enterocolitis which is more a problem of preterm infants whereas severe birth asphyxia is more common in term and near-term infants [37]. We only examined immediate occurring changes and injury in brain tissue not knowing to what extent molecular repair systems would be capable of coping with the produced injury. We used EEG recordings to measure how the lambs coped with the produced brain injury. However, the two-channel EEG represents a basic surveillance tool for brain activity which is also used in the clinical scenario but which is not fully comparable with results gained from a full EEG [38].

Finally, an often-mentioned concern associated with the use of propofol in neonates is the occurrence of the propofol infusion syndrome (PRIS). This is characterized by hemodynamic abnormalities, lactic acidosis, and rhabdomyolysis and carries a mortality of approximately 85% [39]. As published previously, the propofol-treated lambs in our study developed no acidosis, no increase in creatine kinase, and no severe hemodynamic disturbances within 8 h [12]. However, we cannot rule out that a longer use of propofol or a higher number of treated individuals would be accompanied with the occurrence of PRIS due to a relatively high inter-individual variability in propofol pharmacokinetics in preterm and term neonates [40].

## Conclusions

We showed that early propofol administration to the maternal-fetal unit during severe fetal asphyxia was associated with less cerebral dysfunction and less activation of the mitochondrial apoptotic pathway in the brains of late preterm lambs. The observed propofol-related EEG improvements seemed to be mediated by its ROS-scavenging properties, an activation of anti-apoptotic agents, such as pSTAT-3 and Bcl-xL, and its inhibitory influence on the NMDA pathway. The feasibility of designing a propofol-based strategy that blocks brain damage without disrupting normal development in humans will have to be further evaluated.
